# Conservation of CYP307 function between low-identity homologs

**DOI:** 10.17912/micropub.biology.002055

**Published:** 2026-04-26

**Authors:** Caitlyn L Perry, Charles Robin

**Affiliations:** 1 School of BioSciences, The University of Melbourne

## Abstract

The CYP307 enzymes are necessary for the synthesis of the arthropod ecdysteroid moulting hormones, but show a notably elevated duplication and loss rate compared to other enzymes acting in the same pathway. We demonstrate that *Drosophila melanogaster Cyp307a1* null homozygotes can be rescued by ubiquitous expression of *Drosophila hydei Cyp307a3* and *Aedes aegypti* *Cyp307b* transgenes, although amino acid identity between *D* . *melanogaster* CYP307A1 and *A* . *aegypti* CYP307B is less than 35%. This evidence of functional conservation across even distantly-related members of the Cyp307 family provides context for interpreting the evolutionary forces driving Cyp307 copy number variation.

**Figure 1. Testing for transgenic rescue of D . melanogaster Cyp307 mutants:** **A)** *Cyp307a1* and *Cyp307a2* act at different stages of development, but both catalyse one or more steps in the conversion of 7-dehydrocholesterol to 2,22,25-trideoxyecdysone (the ‘Black Box’). We tested the capacity of *D* . *hydei Cyp307a3* and *A* . *aegypti Cyp307b* to rescue *Cyp307a1* and *Cyp307a2* knockouts. **B)** A tree of Cyp307 genes overlaid on the species tree of the three species examined in this study. Circles containing ‘D’ indicate gene duplications; crosses at the end of lines indicate gene losses. **C)** A table of expected outcomes in our crossing scheme; the ubiquitous actin driver (act:GAL4) and transgene constructs are both on chromosome II, while both *Cyp307a1* and *Cyp307a2* are on chromosome III. Survival of non-Curly, non-Tubby adults indicates that transgene expression can rescue homozygous *Cyp307a1* or *Cyp307a2* null mutants. Figure created using BioRender (https://biorender.com) and Inkscape (https://inkscape.org).

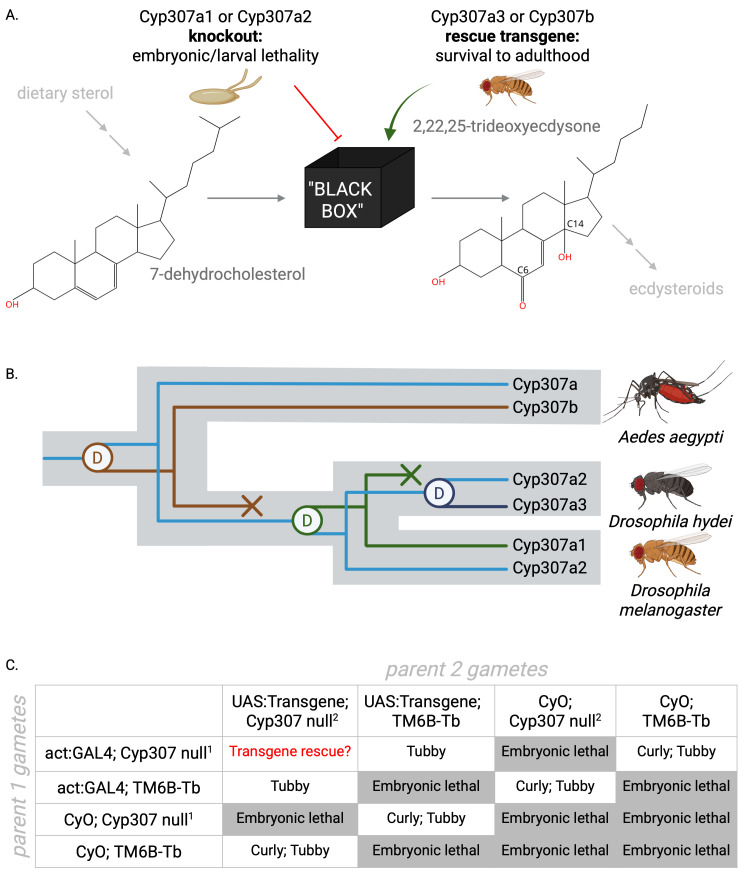



## Description

Arthropod moulting hormone synthesis involves the activity of numerous cytochrome P450s (hereafter P450s), most of which are well characterised functionally (Warren et al., 2002; Petryk et al., 2003; Warren et al., 2004). The Cyp307 group presents a notable exception; while their typically lethal null mutant phenotype indicates that these enzymes are required for ecdysteroid synthesis, they appear to act within a stage of this process known as the ‘Black Box’ (Gilbert et al., 2002; Ono et al., 2006). This name reflects the instability of the intermediates lying between 7-dehydrocholesterol and 2,22,25-trideoxyecdysone (Figure 1a); while a plausible sketch of the Black Box reactions has been made (Pan et al., 2021), it has not been possible to test the activity of any Black Box enzymes directly.

In some cases, it is possible to evaluate likely roles for Black Box enzymes by considering their relatives. For example, Shroud, well established as a Black Box enzyme (Niwa et al., 2010), belongs to the short-chain dehydrogenase/reductase family, some members of which act on steroid hormone hydroxyl groups (e.g. several human short-chain dehydrogenase/reductases, summarised in Bray et al., 2009). As only one dehydrogenation reaction occurs in the Black Box, it is reasonable to infer that Shroud catalyses it. However, two hydroxyl groups are added to the steroid molecule (at C6 and C14; Figure 1a) in the Black Box; CYP307 is likely a catalyst for one or even both reactions, but there is no obvious way to determine which.

A second puzzle surrounding the Cyp307 group is its high rate of gene duplication. The split between the Cyp307a and Cyp307b groups appears to be ancient (Dermauw et al., 2020; Figure 1b); both are present in many modern insect taxa, although loss of one lineage is also common (e.g. Rewitz et al., 2007). More recent Cyp307 duplications are also known, e.g. two separate rounds of Cyp307a duplication in the *Drosophila* genus (Ono *et al* ., 2006; Sztal et al., 2007). We sought to determine whether Cyp307 duplications have resulted in functional differentiation by attempting rescue of *Drosophila melanogaster Cyp307a1* (also called *spook* ) and *Cyp307a2* ( *spookier* ) null mutants with other Cyp307 transgenes (Figure 1a).

We found that ubiquitous expression of *Drosophila hydei Cyp307a3* (which derives from a distinct duplication event to the *D* . *melanogaster Cyp307a1-Cyp307a2* split; Sztal et al., 2007) rescues both *Cyp307a1* and *Cyp307a2* homozygous nulls. Given the lethality of homozygotes for either the CyO or TM6B-Tb balancer chromosomes, complete rescue would result in one in seven (~14%) adult offspring having an act:GAL4/UAS:Transgene; Cyp307 null ^1^ /Cyp307 null ^2^ genotype (indicated by a non-Curly, non-Tubby phenotype; Figure 1c). We observed that 57 of 1203 offspring (~5%) from the *Cyp307a1* rescue cross and 20 of 174 offspring (~11%) from the *Cyp307a2* rescue cross were non-Curly and non-Tubby. While the latter result is consistent (p = 0.5232; two-tailed Fisher’s exact test) with complete rescue, the former indicates only partial rescue (p = 6.448e ^-16^ ; two-tailed Fisher’s exact test); the reason for this discrepancy is unclear, though it is possible that the act:GAL4 construct does not consistently drive transgene expression at levels comparable to endogenous *Cyp307a1* in all relevant tissues. Sequencing the *Cyp307a1* locus from a fly derived by intercrossing two non-Curly, non-Tubby offspring of the *Cyp307a1* rescue cross indicated that only the G481E spo ^1^ allele (Namiki et al., 2005) was present, confirming rescue.

The *Cyp307a1* rescue cross also produced five Curly, non-Tubby offspring, inconsistent with expected cross outcomes (Figure 1c). Survival of homozygous *Cyp307a1* null mutants despite the presence of only one of the driver or the Cyp307 transgene might be explained by maternal protein deposition (of CYP307A1 or GAL4) or ‘leaky’ expression of the Cyp307 transgene rescuing a small proportion of such individuals through embryonic development (note that *Cyp307a1* is not expressed in larval or pupal stages; Ono et al., 2006). Attempts to intercross these Curly, non-Tubby flies failed to produce offspring, making further investigation difficult but perhaps also suggesting that the female reproductive role of *Cyp307a1* (Ono et al., 2006) was not rescued, consistent with the hypothesis of maternal deposition.

Given the capacity of *D* . *hydei Cyp307a3* to rescue both *D* . *melanogaster* Cyp307a paralogs, we tested *Aedes aegypti Cyp307b* only with *D* . *melanogaster Cyp307a1* mutants. We found that 50 of 331 offspring (~15%) were non-Curly, non-Tubby (consistent with complete rescue, p = 0.8261; two-tailed Fisher’s exact test). As with the previous rescue, non-Curly, non-TM6 offspring were successfully crossed, and sequencing of F2 offspring of non-Curly, non-Tubby flies indicated the presence of only the G481E spo ^1^ allele, indicating that *Cyp307b* can also compensate for loss of *Cyp307a1* .

Alongside the Cyp307 duplications mentioned above, we know of duplications in aphids (Christaens et al., 2010), barnacles, horseshoe crabs and springtails (Dermauw et al., 2020), as well as several additional duplications within flies (Perry, 2022). Duplications of other ecdysteroid synthesis enzymes are comparatively rare (Perry, 2022), suggesting Cyp307 duplications confer some specific advantage. While the possibility that the catalytic activity of CYP307 enzymes is rate-limiting for ecdysteroid synthesis (Lafont, 2000; Niwa et al., 2010) may be relevant, determining whether Cyp307 duplications have been followed by functional diversification of the encoded enzymes is essential for fully understanding the evolutionary pressures acting on the Cyp307 family. The capacity of both closely- ( *D* . *hydei Cyp307a3* ) and distantly- ( *A. aegypti* *Cyp307b* ) related members of this family to substitute for *D* . *melanogaster Cyp307a1* indicates broad conservation of function, consistent with earlier findings that Cyp307 paralogs often have non-overlapping expression patterns (e.g. Ono et al., 2006; Sztal et al., 2007, Hentze et al., 2013). It is of course possible that there are minor functional differences between these paralogs (e.g. in substrate preference) which are difficult to detect when using a strong semi-ubiquitous driver. Further testing (e.g. using tissue-specific drivers, determining whether rescue is equally effective with a range of dietary sterols) will be needed for a more granular analysis of Cyp307 functional diversity.

## Methods

Plasmids containing the P450 sequences to be functionally characterised were produced by PCR (using GoTaq® Green Master Mix [Promega; Madison, WI]) of the *Cyp307a3* coding sequence from *Drosophila hydei* and ligation into pUAST-attB (Bischof et al., 2007) and synthesis of a codon-optimised *Aedes aegypti Cyp307b* sequence and ligation into pUC-IDT by Integrated DNA Technologies, Inc. (Coralville, IA). Codon optimisation was performed using Integrated DNA Technologies, Inc.’s Codon Optimization Tool (https://sg.idtdna.com/pages/tools/codon-optimization-tool). Injections of these plasmids into a *D* . *melanogaster* line expressing φ31 integrase were performed by Bestgene Inc. (Chino Hills, CA).

Expression of transgenes was driven by actin-GAL4 (Ito et al., 1997), as a phantom-GAL4 driver (Guittard et al., 2011) intended to better recapitulate endogenous expression of the Cyp307 genes proved unstable (as determined by induction of fluorescence in a UAS-GFP line). Both the transgenic P450 construct and the actin-GAL4 driver were held over a CyO balancer, and both Cyp307 null alleles were held over a TM6B-Tb balancer (e.g. act:GAL4/CyO; spo ^1^ /TM6B-Tb x UAS- *D* . *hydei* - *Cyp307a3* /CyO, Df(3L)Exel6105/TM6B-Tb; Figure 1c). We expect absence of the Tubby phenotype (indicating a compound *Cyp307a1* or *Cyp307a2* null) to be viable only in the presence of both driver and transgene (i.e. absence of the Curly phenotype). PCR to confirm absence of wild-type *Cyp307a1* used the primers CGACGATTTTCGAGGTGCTG (forward) and ATGGAAAACAATCGGCAGGC (reverse). Sanger sequencing was performed by the Australian Genome Research Facility (Melbourne, Australia). Fisher’s exact tests were performed in R (version 4.3.2; R Core Team, 2025).

## Reagents

**Table:** 

**Reagent type**	**Genotype**	**Description**	**Available from:**
Fly strain	y[1] M{RFP[3xP3.PB] GFP[E.3xP3]=vas-int.Dm}ZH-2A w[*]\int.NLS}X; P{UAS:DhCyp307a3}attP40	*Cyp307a3* rescue	Robin lab
Fly strain	y[1] M{RFP[3xP3.PB] GFP[E.3xP3]=vas-int.Dm}ZH-2A w[*]\int.NLS}X; P{UAS:AeCyp307b}attP40	*Cyp307b* rescue	Robin lab
Fly strain	w[*]; If/CyO; Sb/TM6, Tb	Double balancer	Robin lab
Fly strain	w[1118]; Df(3L)Exel6105, P{w[+mC]=XP-U}Exel6105/TM6B, Tb[1]	*Cyp307a1* ( *spook* ) deficiency	RRID:BDSC_7584
Fly strain	spo[1] st[1] e[1]/TM3, Sb[1]	*Cyp307a1* ( *spook* ) null allele&nbsp;	RRID:BDSC_3276
Fly strain	w[*]; spok[e-204] e[1]/TM3, P{w[+mC]=GAL4-twi.G}2.3, P{UAS-2xEGFP}AH2.3, Sb[1] Ser[1]	*Cyp307a2* ( *spookier* ) null allele	RRID:BDSC_82442
Fly strain	w[*]; st[1] spok[Z712]/TM3, P{w[+mC]=ActGFP}JMR2, Ser[1]	*Cyp307a2* ( *spookier* ) null allele	RRID:BDSC_82443
Fly strain	y[1] w[*]; P{w[+mC]=Act5C-GAL4}25FO1/CyO, y[+]	Actin-Gal4 driver	RRID:BDSC_4414

*spo ^1^* and Df(3L)Exel6105 (Ryder, 2004) mutants served as *Cyp307a1* nulls; we favoured the use of compound heterozygotes to reduce the likelihood of undocumented lethal mutations in other genes confounding analysis of the crosses. *spok* ^e-204^ and *spok ^Z712^* (Koundakjian et al., 2004; Honda, 2019) were used as *Cyp307a2* nulls. All stocks were reared at 25°C on molasses media.

## References

[R1] Bischof J, Maeda RK, Hediger M, Karch F, Basler K (2007). An optimized transgenesis system for Drosophila using germ-line-specific phiC31 integrases.. Proc Natl Acad Sci U S A.

[R2] Bray JE, Marsden BD, Oppermann U (2008). The human short-chain dehydrogenase/reductase (SDR) superfamily: a bioinformatics summary.. Chem Biol Interact.

[R3] Dermauw W, Van Leeuwen T, Feyereisen R (2020). Diversity and evolution of the P450 family in arthropods.. Insect Biochem Mol Biol.

[R4] Gilbert LI, Rybczynski R, Warren JT (2002). Control and biochemical nature of the ecdysteroidogenic pathway.. Annu Rev Entomol.

[R5] Hentze JL, Moeller ME, Jørgensen AF, Bengtsson MS, Bordoy AM, Warren JT, Gilbert LI, Andersen O, Rewitz KF (2013). Accessory gland as a site for prothoracicotropic hormone controlled ecdysone synthesis in adult male insects.. PLoS One.

[R6] Honda, B. (2019). Honda alleles.&nbsp;FlyBase ID: FBrf0242880. https://flybase.org/reports/FBrf0242880

[R7] Koundakjian EJ, Cowan DM, Hardy RW, Becker AH (2004). The Zuker collection: a resource for the analysis of autosomal gene function in Drosophila melanogaster.. Genetics.

[R8] Moeller ME, Nagy S, Gerlach SU, Soegaard KC, Danielsen ET, Texada MJ, Rewitz KF (2017). Warts Signaling Controls Organ and Body Growth through Regulation of Ecdysone.. Curr Biol.

[R9] Namiki T, Niwa R, Sakudoh T, Shirai K, Takeuchi H, Kataoka H (2005). Cytochrome P450 CYP307A1/Spook: a regulator for ecdysone synthesis in insects.. Biochem Biophys Res Commun.

[R10] Niwa R, Namiki T, Ito K, Shimada-Niwa Y, Kiuchi M, Kawaoka S, Kayukawa T, Banno Y, Fujimoto Y, Shigenobu S, Kobayashi S, Shimada T, Katsuma S, Shinoda T (2010). Non-molting glossy/shroud encodes a short-chain dehydrogenase/reductase that functions in the 'Black Box' of the ecdysteroid biosynthesis pathway.. Development.

[R11] Ono H, Rewitz KF, Shinoda T, Itoyama K, Petryk A, Rybczynski R, Jarcho M, Warren JT, Marqués G, Shimell MJ, Gilbert LI, O'Connor MB (2006). Spook and Spookier code for stage-specific components of the ecdysone biosynthetic pathway in Diptera.. Dev Biol.

[R12] Pan X, Connacher RP, O'Connor MB (2020). Control of the insect metamorphic transition by ecdysteroid production and secretion.. Curr Opin Insect Sci.

[R13] Perry C. 2022. Comparative genetics of invertebrate moulting [dissertation]. [Melbourne (Australia)]: The University of Melbourne. (Perry 2022) https://minerva-access.unimelb.edu.au/handle/11343/312568

[R14] Petryk A, Warren JT, Marqués G, Jarcho MP, Gilbert LI, Kahler J, Parvy JP, Li Y, Dauphin-Villemant C, O'Connor MB (2003). Shade is the Drosophila P450 enzyme that mediates the hydroxylation of ecdysone to the steroid insect molting hormone 20-hydroxyecdysone.. Proc Natl Acad Sci U S A.

[R15] R Core Team (2025). R: A Language and Environment for Statistical Computing. R Foundation for Statistical Computing, Vienna, Austria. https://www.R-project.org/

[R16] Rewitz KF, O'Connor MB, Gilbert LI (2007). Molecular evolution of the insect Halloween family of cytochrome P450s: phylogeny, gene organization and functional conservation.. Insect Biochem Mol Biol.

[R17] Ryder, E.J. (2004). Exelixis CG deletion data.&nbsp;FlyBase ID: FBrf0184335. https://flybase.org/reports/FBrf0184335

[R18] Sztal T, Chung H, Gramzow L, Daborn PJ, Batterham P, Robin C (2007). Two independent duplications forming the Cyp307a genes in Drosophila.. Insect Biochem Mol Biol.

[R19] Warren JT, Petryk A, Marqués G, Parvy JP, Shinoda T, Itoyama K, Kobayashi J, Jarcho M, Li Y, O'Connor MB, Dauphin-Villemant C, Gilbert LI (2004). Phantom encodes the 25-hydroxylase of Drosophila melanogaster and Bombyx mori: a P450 enzyme critical in ecdysone biosynthesis.. Insect Biochem Mol Biol.

